# The TEAM instrument for measuring emergency team performance: validation of the Swedish version at two emergency departments

**DOI:** 10.1186/s13049-021-00952-9

**Published:** 2021-09-20

**Authors:** Klas Karlgren, Anders Dahlström, Anderz Birkestam, Annelie Drevstam Norling, Gustav Forss, Mikael Andersson Franko, Simon Cooper, Thomas Leijon, Charlotta Paulsson

**Affiliations:** 1 Department of Research, Education, Development and Innovation, Södersjukhuset, 118 83 Stockholm, Sweden; 2 MINT, Department Learning, Informatics, Management and Ethics, Karolinska Institutet, 171 77 Stockholm, Sweden; 3 Faculty of Health and Social Sciences, Western Norway University of Applied Sciences, 5063 Bergen, Norway; 4 Department of Neonatology, Sachs’ Children and Youth Hospital, Stockholm, Sweden; 5 Department of Emergency, 118 83 Södersjukhuset, Stockholm, Sweden; 6 Department of Clinical Science and Education, Södersjukhuset, Karolinska Institutet, Södersjukhuset, 118 83 Stockholm, Sweden; 7 Health Innovation and Transformation Centre (HITC), School of Health, Federation University Australia, Room 113, Building 1, Berwick Campus, Clyde Road, Berwick, VIC 3922 Australia; 8 Department of Emergency Medicine, Capio S:t Göran’s Hospital , 112 81 Stockholm, Sweden

**Keywords:** Teamwork, Communication, Assessment, Instrument, Validation, Measure, Non-technical skills, Observational research, Emergency

## Abstract

**Background:**

The Team Emergency Assessment Measure (TEAM) questionnaire is designed for rating the non-technical performance of emergency medical teams during emergencies, e.g., resuscitation or trauma management. Originally developed in Australia it has today been translated and validated into eleven languages, but a Swedish version is lacking. The aim was therefore to cross-culturally translate and evaluate the reliability and validity of the TEAM questionnaire in a Swedish health care setting.

**Methods:**

The instrument was forward and backward translated and adapted into a Swedish context according to established guidelines for cross-cultural adaptation of survey-based measures. The translated version was tested through 78 pairwise assessments of 39 high-priority codes at the emergency departments of two major hospitals. The raters observed the teams at work in real time and filled in the questionnaires immediately afterwards independently of each other. Psychometric properties of the instrument were evaluated.

**Results:**

The original instrument was translated by pairs of translators independently of each other and reviewed by an expert committee of researchers, nurses and physicians from different specialties, a linguist and one of the original developers of the tool. A few adaptations were needed for the Swedish context. A principal component factor analysis confirmed a single ‘teamwork’ construct in line with the original instrument. The Swedish version showed excellent reliability with a Cronbach’s alpha of 0.955 and a mean inter-item correlation of 0.691. The mean item-scale correlation of 0.82 indicated high internal consistency reliability. Inter-rater reliability was measured by intraclass correlation and was 0.74 for the global score indicating good reliability. Individual items ranged between 0.52 and 0.88. No floor effects but ceiling effects were noted. Finally, teams displaying clear closed-loop communication had higher TEAM scores than teams with less clear communication.

**Conclusions:**

Real time observations of authentic, high priority cases at two emergency departments show that the Swedish version of the TEAM instrument has good psychometric properties for evaluating team performance. The TEAM instrument is thus a welcome tool for assessing non-technical skills of emergency medical teams.

## Background

Effective interprofessional teamwork and team communication are crucial in the delivery of safe, high-quality health care [1–3], with adverse outcomes often ascribed to inadequate leadership, communication, teamwork, decision-making and task distribution [4–6]. Team collaboration and communication, especially in critical situations, may be challenged by factors such as heavy workloads requiring multi-tasking and dealing with numerous patients, hasty decisions based on incomplete information, dynamic teams with multiple hand-overs and frequent staff member substitutions, lack of resources, interruptions, and background noise [1, 2, 7].

To manage complex tasks, teams have to be able to exchange information, coordinate efforts and adapt to changing situational factors. Teamwork has been described as the interrelated team member behaviors, thoughts, and feelings needed for the team to function as a unit relying on mechanisms such as closed-loop communication, shared understanding, and mutual trust [8, 9]. A current discussion, not only in health care, is how education and training can better prepare for the requirements of workplaces [10]. Continual efforts have been made to define and assess individual and collective competence relating to collaboration, teamwork and interprofessional interaction [11, 12]. Despite having a comparatively strong tradition of emphasizing teamwork in health care in Scandinavia, the need for a greater focus on teamwork has been called for both in civilian health care [13] and military medicine [14]. The Swedish Society of Medicine and the Swedish Society of Nursing have jointly argued for more team training in health care education [15]. Besides more team training, physicians have argued for the need to investigate how non-technical skills develop and how team training contributes to clinical work [16]. A recent review notes that team research has received greater attention in the area of health care over the last decade but has nevertheless resulted in a small number of research studies [3]. The review concludes that this may relate to the difficulties in quantifying teamwork.

Standardized observation in the emergency department is one method that may be helpful in identifying threats to safety and opportunities for improvement [17]. Observations can provide insights about teamwork on a level that is unmatched by other methods; interview or focus group participants are for example unlikely to remember or be able to describe with precision the details that observers can detect [18]. However, learning to observe and analyze the performance of medical teams is not a trivial task and may not develop with experience alone. While experienced simulation educators may display skills in analyzing team performance [19], such skills may be the result of years of competence development [20].

The complexities of team performance pose a challenge to measuring team performance [21] and a challenge for research is to develop and validate instruments for team performance assessment both for the design of team training efforts as well as effective clinical work [22]. A number of assessment tools have been developed for measuring the teamwork performance in crisis situations addressing various aspects of non-technical skills such as leadership, communication, coordination, situation awareness, planning, re-evaluation, and task and resource management [23–26]. Tools have most often been assessed within emergency medicine teams followed by obstetrical and pediatric teams and in most cases the measurement properties of the tools have been assessed based on simulated cases [25]. Many of the available assessment tools overlap regarding the skills that are assessed and the need for more psychometric evidence of the properties of assessment tools has been argued for [24]. While the choice of instrument will depend on many parameters such as the context and goal of the assessment, a recent review of assessment measures singled out the Team Emergency Assessment Measure (TEAM) as the most promising tool as it has shown robust evidence of its measurement properties [25].

TEAM is an observational tool for the assessment of resuscitation and emergency team performance which addresses three overall categories of teamwork—leadership, teamwork and task management—covering leadership control; communication; co-operation and co-ordination; team climate; adaptability; situation awareness; prioritization; and clinical standards. In addition, one item is a global rating measuring the overall ‘gut reaction’ to performance. The original English version of the instrument has been translated into 11 languages including Chinese, Finnish [27], French [28], German [29], Hebrew, Italian, Persian and Portuguese.^1^

A Swedish version of the TEAM instrument has however been lacking. The aim of this study was therefore to cross-culturally translate and evaluate the reliability and validity of the Team Emergency Assessment Measure (TEAM) questionnaire in a Swedish health care setting.

## Methods

The original TEAM instrument was translated into Swedish following the methods suggested for cross-culturally translating an instrument and adapting it to another cultural context [30–32]. Translation of the instrument is considered the first step, but different models have been proposed for taking into account cultural, idiomatic, conceptual, experiential, and contextual aspects to minimize risks for biases and detect ambiguous wordings [31, 33]. Instruments which are not accurately adapted risk generating inconsistent and unreliable data [31]. The suggested multi-step procedures for translating and adapting instruments need to balance the needs for ensuring that a translated instrument on the one hand reflects the content of the original version and at the same time makes sense in the target language in a new context.

**Fig. 1:**
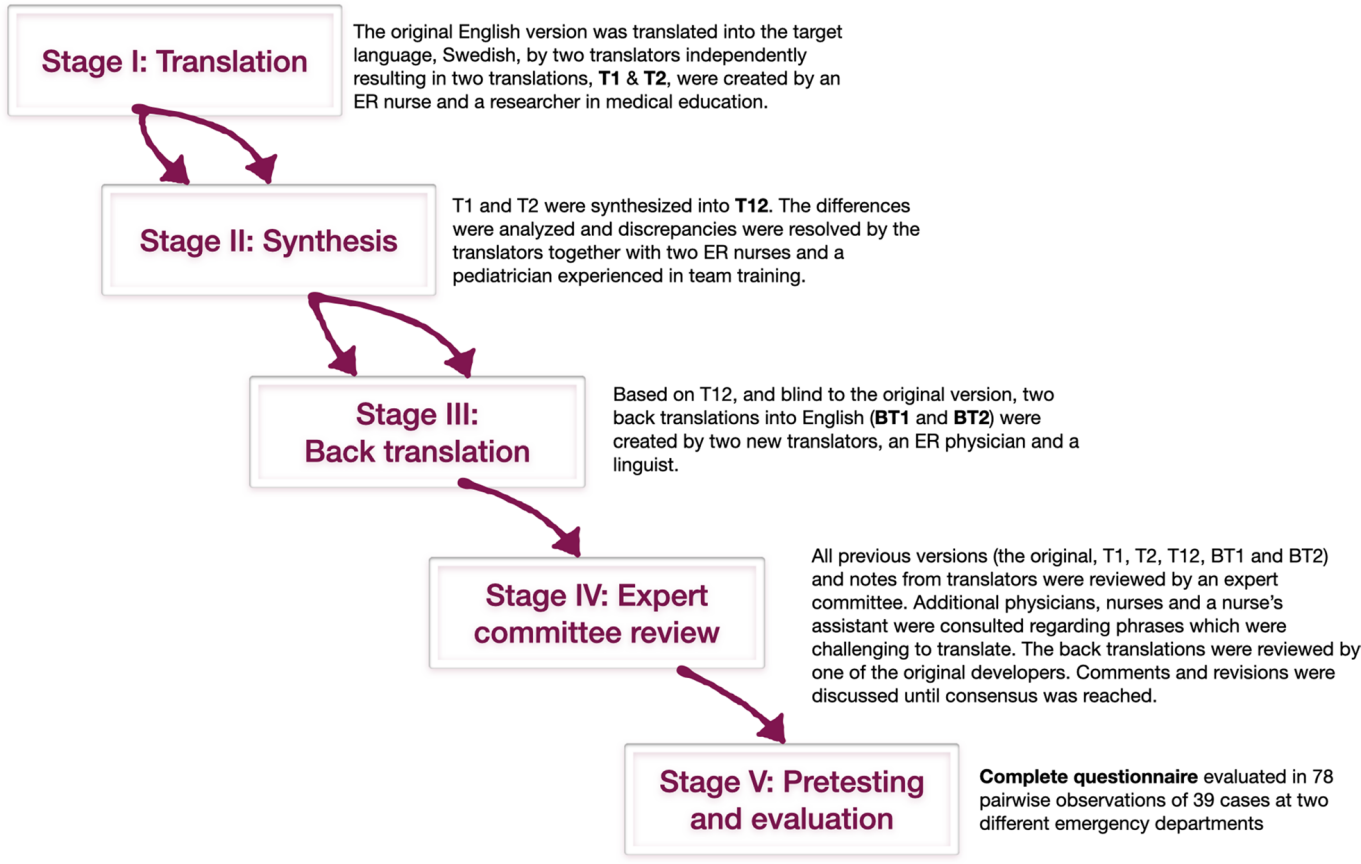
The cross-cultural translation and adaptation process

First two translators independently of each other translated the original instrument into the target language, Swedish (see Fig. 1). One of the forward translations (T1) was completed by an experienced nurse working at a large emergency room and the other by a researcher in medical education (T2). Both translators were knowledgeable of the topic. To synthesize the results, two nurse specialists in emergency nursing and a pediatrician experienced in team training were invited to discuss the differences between translations. Issues were resolved through consensus and a common translation (T-12) was produced.

Using the T-12 version of the questionnaire and blinded to the original version, two new translators translated back from Swedish into English enabling comparisons of the back-translations (BT1 and BT2) to the original version. One of the back-translators was a Swedish emergency physician and professor of emergency medicine with English as her mother tongue while the other was a Swedish professor and linguist with extensive work experience from the United States. The two translators worked independently of each other and were not informed of the concepts explored to avoid information bias and to elicit unexpected meanings of the items [30, 33].

**Fig. 2:**
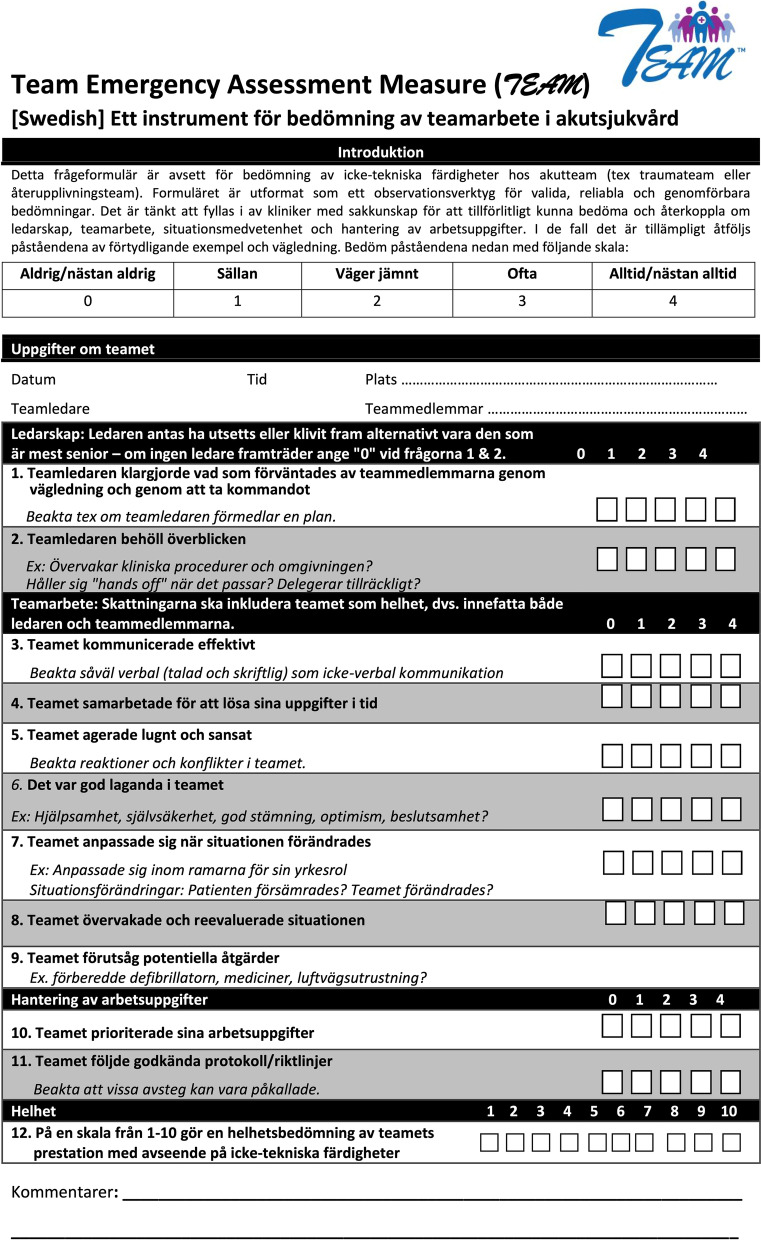
The Swedish version of the Team Emergency Assessment Measure (TEAM) questionnaire. The instrument and instructions for its use can be found here: http://medicalemergencyteam.com

The different translations of each item and the translators’ notes were color-coded and compiled in one document for easier comparison of the different versions. An expert committee consisting of the translators and the two additional ER nurses and the pediatrician reviewed any discrepancies to create a new synthesis. As certain words and phrases were challenging to translate, the committee consulted another senior pediatrician and simulation educator, an intensive care nurse, a pediatric nurse, two pre-hospital care nurses, a surgeon, a nurse’s assistant, and a nurse specialist in emergency nursing responsible for the hospital’s CPR training. Involving the original instrument’s authors has been recommended [31] and the back-translations were therefore reviewed by Professor Simon Cooper, the lead developer of the original English instrument. New comments were discussed until reaching consensus and finally resulting in a Swedish version (Fig. 2). The Swedish translation was then tested and evaluated as described below.

### Cases and participants

Pairwise observers made 78 real time observations of 39 cases at the emergency departments in two hospitals in Stockholm: 13 cases at the Saint Göran Hospital and 26 cases at the Södersjukhuset hospital. Recommendations about sample numbers required for validating an instrument vary to a high degree and an absolute rule does not exist [32, 34]. A previous study validating a French translation of the same instrument using video-recordings of cardiac arrest simulations served as inspiration [28]. However, as this study investigated authentic cases without being able to predict the kind of patient cases, which weekday or time of the day that they occurred, nor who would be members and leaders of the teams the number of observed cases was increased. The hospitals that were included are both major hospitals in Stockholm and the Södersjukhuset hospital has one of the largest emergency departments in Scandinavia. The observed cases were the most highly prioritized emergency codes (red, prio-1) involving life-threatening injuries and conditions. The cases included conditions and symptoms such as: arrhythmia, abdominal pains, allergic reactions, cardiac arrest, chest pains, dyspnea, exacerbations of chronic obstructive pulmonary disease, injuries from falling and traffic accidents, intoxications, loss of consciousness, presyncope, sepsis, and stroke. The patients typically arrived by ambulance but may also have checked in at the receptionist’s desk or been transferred from other departments within the hospital. These patients were then managed by an emergency team. The observed teams had between four to seventeen members (mean = 7; median = 6) with a typical team consisting of one-two physicians, two nurses, one nurse’s assistant and a medical student. Often an anesthesiologist and an anesthesia nurse were also present and occasionally a surgeon, cardiologist or other specialist as well as more nurses including stroke nurses.

### Data collection

The observers were experienced nurses and physicians employed at the two emergency departments and had practiced using the questionnaire on video-recordings of simulated cases equivalent to the authentic ones. The observers were allowed time off from their ordinary work tasks and they were thus not expected to be involved in regular work when rating. One pair of observers rated all cases at the Saint Göran hospital. At the Södersjukhuset hospital pairwise observations were performed with one person conducting all observations together with one other person from a pool of four observers. Altogether 78 observations were conducted. The Swedish version of the TEAM questionnaire was filled in independently of each other immediately after having observed the cases. The durations of the observations ranged between 7 and 56 min (mean = 25 min 27 s; median = 22 min 25 s). Observations were conducted during different weekdays and different times of the day including nighttime to avoid biases related to time and day. The data collection was initiated in 2015 and completed in 2020.

### Data analysis

The following metrics were analyzed: (1) A principal component factor analysis; (2) internal consistency among items was analyzed by calculating Cronbach’s alpha coefficients; (3) internal consistency was also analyzed by investigating inter-item correlation; (4) item-scale correlation; (5) inter-rater reliability of the pairwise observers’ assessments using Intra Class Correlation (ICC); (6) floor and ceiling effects; and finally, (7) a Kruskal–Wallis test was used to investigate if the quality of the teams’ communication was reflected in the results of the TEAM instrument. Data from a study performed at the Södersjukhuset hospital which had a focus on closed-loop communication and other non-technical skills were used for comparison; the same team situations were assessed regarding the quality of communication using a software designed for live observations (Obansys). Raters used a predefined observation schedule to count each occurrence of a (1) clear, directed order (defined as spoken out load for the entire team to hear and clearly addressed to another team member by name), (2) unclear order, (3) clear confirmation (clearly responding to an order by repeating it), and (4) unclear or missing confirmation. Counting was done by clicking on corresponding buttons in the Obansys app on an iPad. The results of these counts were not available for the observers, but the measures were later used to categorize the quality of the communications as being either low, intermediate or high based on an overall assessment of the four measures. The quality of communication was interpreted as high if at least three of the four measures were above the medians, or, if two scores were above the medians and none of the other two measures were below median scores. Low quality was rated in the opposite fashion. All other cases—which thus did not consistently show low or high scores—were considered intermediate. *P* values of < 0.05 were considered statistically significant.

We hypothesized that TEAM scores would differ according to the quality of communication with cases of high quality receiving higher scores.

Analyses were performed using R (v3.6.0, 2021, Vienna, Austria) and Microsoft Excel v16.46 for Mac.

## Results

### Cross-cultural translation and adaptation

While the pairs of translators occasionally suggested different wordings and phrases a consensus was reached on a final translation that followed the original version but which was adapted to the Swedish health care context. Finding a satisfactory translation for one phrase was however challenging. A literal translation of the first item about the team leader (*The team leader let the team know what was expected of them through direction and command*) was not applicable in the Swedish setting. The phrase”through command” was considered to have an authoritarian or militaristic connotation and being interpreted negatively in a civilian health care setting and as a marker of unsound leadership, in contrast to what was intended by the original item.

The expert committee therefore consulted numerous physicians and nurses within different specialties as well as experienced simulation and CPR educators to find a better wording. Almost 20 different Swedish translations were tried (‘befallningar’, ‘order’, ‘kommandon’, ‘uppmaningar’ etc.) but the conclusion was that the difficulty was less about identifying the perfect literal translation but more about cultural differences regarding leadership. This tension between a literal translation and a cultural bias was resolved by consulting experienced simulation educators about how they teach team leaders with regard to leadership processes in relation to such items as “let the team know what is expected of them”. A wide-spread team-training and simulation approach in Sweden—the CEPS model (Concept for Patient Simulation)—emphasizes that team leaders should convey a plan to the team rather than to simply give orders. By making the team aware of the team leader’s plan, team members have a better chance of understanding the team leader’s instructions and what is expected of them. Taking this into account, the final solution to the translation challenge was to use a literal translation but also to add a prompt after the item relating to the Swedish practice (“Consider whether the team leader conveys a plan”). This solution thus follows the original version but lessens the risk of misunderstanding the item as something negative. The original author (SC) of the TEAM instrument suggested seven changes. Most of these concerned inaccuracies and vagueness in the back-translations. Minor modifications were made to the title and background information but changes to the actual items were not needed.

### Psychometric results

A principal component factor analysis was conducted and identified one component explaining 73.6% of the variance which is in line with the original instrument that was shown to measure a single factor, ‘teamwork’ [35].

Internal consistency or scale reliability, i.e., the extent to which all the items in a test measure the same concept, was measured by calculating Cronbach’s alpha and was 0.955 for the entire Swedish TEAM instrument thus confirming excellent internal consistency. To further investigate internal consistency inter-item correlations were also calculated. The extent to which scores on one item were related to scores on all the other items in the instrument were examined showing a range of 0.43–0.86 and a mean correlation of 0.691 which is considered high. In comparison, the inter-item-correlations of the original instrument were 0.58–1 and of the French translation 0.47–0.85 [28].

An item-scale (item-total) analysis was performed to check if any item was inconsistent with the averaged behavior of the others. Each item was correlated with the total scale score (based on all items except nr 12) corrected for overlap, i.e., the particular item taken out of the total score. The item-scale correlations ranged between 0.70 and 0.89 with a mean of 0.82 which is considered high and indicating that items measure the same general construct and thus implying a high internal consistency reliability, see Table 1.

**Table 1:** Item-scale correlations, inter-rater reliabilities (IRR) measured using intraclass correlation (ICC), % of lowest and highest values to indicate floor and ceiling effects, and *p* values of the Kruskal–Wallis test

Item	Cronbach’s alpha	Item-scale correlation	IRR (ICC)	Floor (%)	Ceiling (%)	Kruskal–Wallis test
1. The team leader let the team know what was expected of them through direction and command	0.79	0.77	0.88	15.4	17.9	0.048*
2. The team leader maintained a global perspective Prompts: monitoring clinical procedures and the environment? Remaining ‘hands off’ as applicable? Appropriate delegation	0.86	0.83	0.67	2.6	41.0	0.035*
3. The team communicated effectively Prompts: verbal, non-verbal and written forms of communication?	0.87	0.89	0.77	3.8	15.4	0.017*
4. The team worked together to complete the tasks in a timely manner	0.87	0.86	0.59	1.3	33.3	0.037*
5. The team acted with composure and control Prompts: applicable emotions? Conflict management issues?	0.84	0.76	0.62	0.0	70.5	0.089
6. The team morale was positive Prompts: appropriate support, confidence, spirit, optimism, determination?	0.86	0.83	0.69	1.3	55.1	0.088
7. The team adapted to changing situations Prompts: adaptation within the roles of their profession? Situation changes: patient deterioration? Team changes?	0.80	0.70	0.76	2.4	22.0	0.349
8. The team monitored and reassessed the situation	0.85	0.83	0.52	3.9	29.9	0.284
9. The team anticipated potential actions Prompts: preparation of defibrillator, drugs, airway equipment?	0.86	0.86	0.52	6.5	31.2	0.081
10. The team prioritised tasks	0.81	0.80	0.60	7.7	28.2	0.018*
11. The team followed approved standards and guidelines Prompt: some deviation may be appropriate?	0.93	0.89	0.60	6.5	31.2	0.022*
12. On a scale of 1–10 give your global rating of the team’s performance	0.94	–	0.83	1.3	3.9	0.012*

*P* values of < 0.05 are marked with a *. The items are presented in English for readability

Inter-rater reliability was measured by calculating intraclass correlation (ICC) of the following type: one-way random effects, absolute agreement, single rater measurement. The ICC measurements of the individual items ranged between 0.52 and 0.88. According to common guidelines [36], less than 0.40 is considered poor, 0.40 and 0.59 fair, 0.60 and 0.74 good and 0.75 and 1.00 excellent. The measurements thus range between fair and excellent and most items were either good or excellent. Two items (8 and 9) resulted in an ICC value of 0.52 and another item (4) resulted in 0.59. As a comparison, the original instrument showed an ICC range of 0.59–0.88.

Inter-rater reliability for the global TEAM score (a sum of the scores of all items) was also calculated (two-way, random effects) showing 0.74 and thus indicating good reliability.

**Fig. 3:**
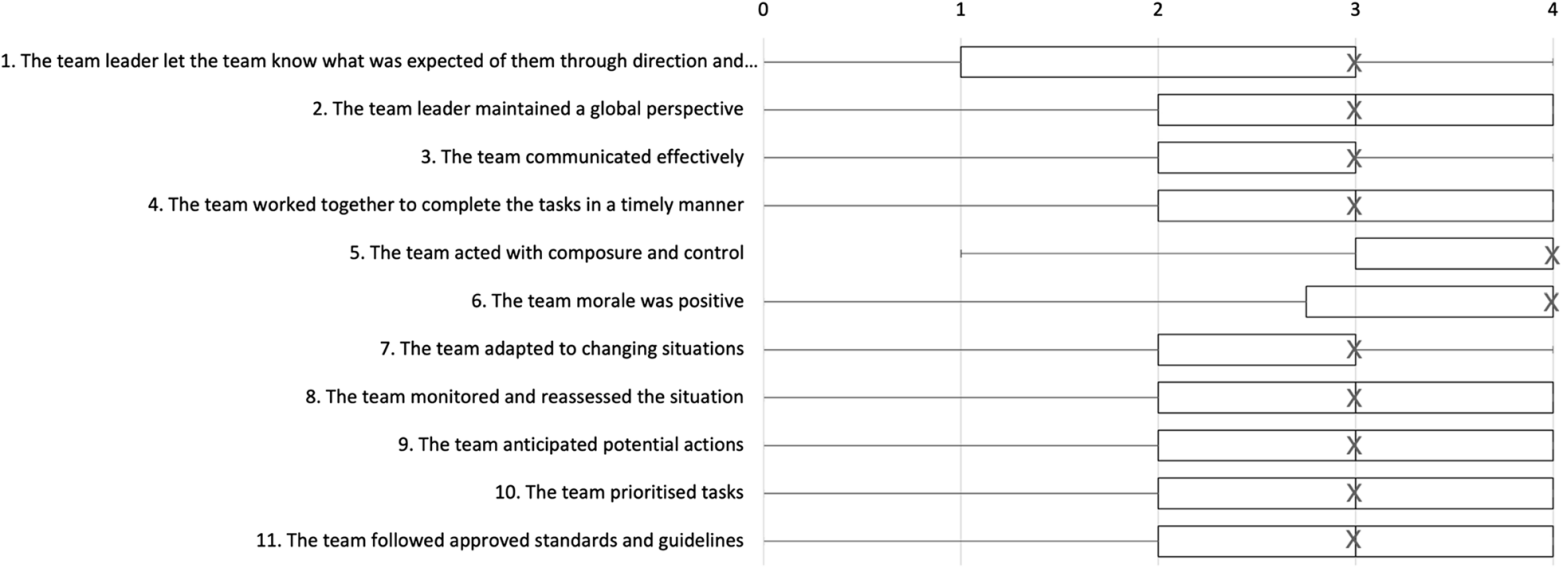
Box plot with interquartile ranges showing the distributions of answers to the TEAM items. The items are presented in English for comprehension. Crosses indicate median scores. In cases 1, 3 and 5–7 the medians and the third quartiles are equal

All scale values of all items were used except for item 5 which never received the lowest score, ‘0’, see Fig. 3. Floor or ceiling effects are considered to be present if more than 15% of cases achieved the lowest or highest possible score. The distribution of the results showed no floor effects except for item 1, see Table 1. However, ceiling effects were noted for all items except for item 12.

**Fig. 4:**
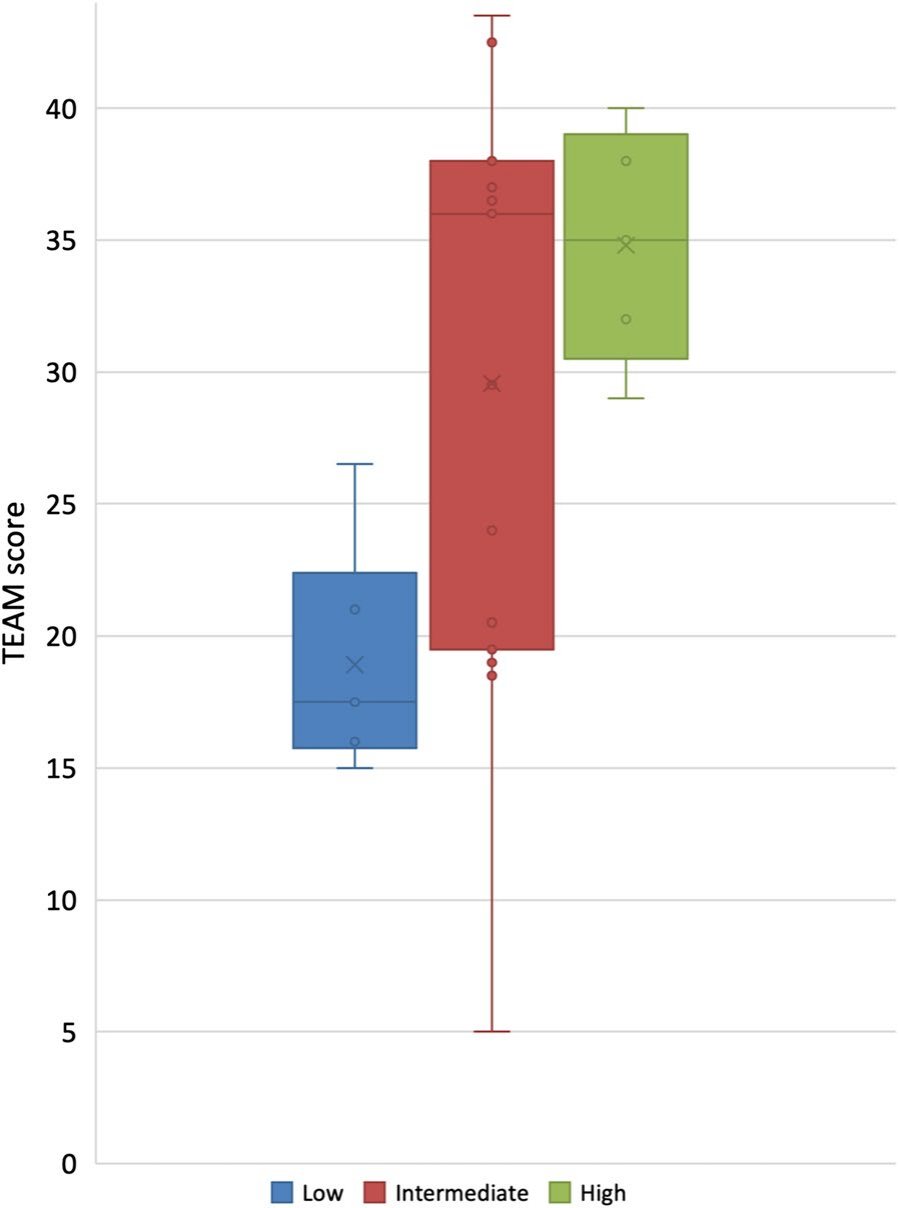
A box plot showing TEAM scores and the interquartile ranges for cases with low, intermediate, and high quality communication. The crosses show mean scores

A Kruskal–Wallis test (one-way ANOVA on ranks) was performed to compare the TEAM results to the quality of communication in the teams. Figure 4 shows the TEAM results of the cases having low, intermediate and high quality communication. As illustrated by the box plots, a statistically significant association was observed between the quality of communication and TEAM scores (*p* = 0.019). The mean, median and range measures were as follows for the three levels of communication: low (*M* = 19, *Mdn* = 18, range = 15–27), intermediate (*M* = 30, *Mdn* = 36, range = 5–44) and high quality (*M* = 35, *Mdn* = 35, range = 29–40). Cooper and colleagues suggest that scores of 33 or less are a ‘poor’ performance, 34–39 a ‘good’ team performance, and 40 and above equates to ‘excellent’ team performance (score range 0–44) [37]. On an item level, the Kruskal–Wallis test showed that the quality of the teams’ communication was significantly associated with seven of the twelve TEAM items, cf. Table 1. Items 1–4 and 10–12 all showed significant associations between the quality of communication and the TEAM items.

## Discussion

The TEAM tool has previously been shown to be a valid, reliable and feasible nontechnical observational tool for the assessment of resuscitation team performance in simulated environments and further evaluation especially in’real’ settings have been called for [28, 35]. Most existing tools available for assessing teamwork performance of teams in crisis situations have been investigated using simulated scenarios and it is therefore unclear whether the measurement properties of the tools are applicable for teamwork assessment in clinical practice [25]. This study contributes with findings from studies of a Swedish version of TEAM in two emergency room settings.

The TEAM instrument was translated and adapted to a Swedish health care context. The pairwise translations occasionally generated divergent wordings and phrases but most of these were rather easily resolved by consensus. Translation—and especially when doing pairwise translations as in this study—often generates unexpected findings due to the vagueness of language or because languages are not semantically aligned [38]. Lexical correspondences do not always exist for all items and it is an essential part of translation to establish them [39]. The translation of one item turned out to be challenging as it resulted in tension between a literal translation and obtaining conceptual equivalence regarding team leadership in the two languages. A balance needed to be found between the goals of strictly following the original version and finding a wording that users in the target context were comfortable with. Translation of instruments sometimes requires altering, adding or even deleting items [40]. Such alterations were avoided by instead adding a prompt that supported interpretation of the item in the intended way and which enabled retaining a literal translation of that item itself. This solution could be a convenient option also in the translation of other instruments across languages and cultures.

This study shows that the Swedish version has similar psychometric properties as the previously validated original version developed in English in 2010 confirming that the translation did not alter the properties of the instrument [35, 37]. One component was identified corresponding to the single factor, ‘teamwork’, found in the original. The findings show that internal consistency and inter-rater reliability of the Swedish version also are consistent with the original. Internal consistency was excellent and inter-item correlations and item-scale analyses were high. The inter-rater reliability of the global TEAM score was also good with individual items ranging between fair and excellent with most items getting either good or excellent results. Ceiling effects were observed and a reason for this may be that the participating teams were well above average when it comes to both competence and experience. The observed teams were teams managing high-priority cases at two of the largest emergency departments in Sweden and even though much variation was observed between cases—all scale values were used with only one exception—their (high) levels of teamwork experience are likely to have generated the disproportionately high scores. Future studies should evaluate the instrument using teams with more varying levels of experience.

Validity was supported by the observation that the Swedish TEAM instrument discriminated between cases of low, intermediate and high quality of communication. Teams displaying clear communication had higher TEAM scores than teams with less clear communication. The communication quality was based on a count of the two key elements of closed-loop communication, the numbers of clear and unclear orders and confirmations. Closed-loop communication is a well-established communication strategy in emergency teams [41] and teams using closed-loop communication are associated with greater clinical efficiency compared to teams with less clear communication [42]. While there admittedly are many more aspects of communication and teamwork than closed-loop communication, the clear association between TEAM results and the clarity of the communication indicates construct validity. Moreover, the categorizations of the communication quality were based on real time counts of well-defined, observable behaviors rather than retrospective assessments or generalizations minimizing the need for interpretation and risks of biases. An alternative approach could have been to investigate how TEAM scores are associated with characteristics of the teams such as training, experience or team composition, but the performance of teams may vary from case to case regardless of proper training, experience or team composition and a strength of this study was that the analyses were based on the teams’ actual performance in these specific cases rather than on some general characteristic.

Observing and assessing leadership, teamwork and team interaction in interprofessional teams in the emergency department is a challenging task. A limitation of this study is that the assessments were done in real-time. In many cases, if possible, it may be preferable to make assessments based on video-recordings as they can be replayed to double-check observations and even scrutinized in slow-motion. Recordings could also enable other types of analyses with other observers. A video-recording also ensures that observers see the same thing which is not possible when performing direct observation. The observers in our study often stood in different ends of the room giving them different views of the teams and events possibly affecting what they observed visually and what they heard. Despite these challenges, inter-rater reliability was good implying that the TEAM instrument is robust enough also for real-time observations. For logistical reasons the same pair of observers could not perform all observations at one of the hospitals which was not optimal and this may have affected the results negatively. The presence of observers in the emergency room could potentially have affected the behaviors of the team members. However, observers (e.g., students) are not uncommon in the emergency room and studies have indicated that the effects of an observer’s presence may be less of threat to validity than commonly imagined [43, 44]. Observer presence effects may be lessened when the researcher is more or less a member of the community being observed [45] and this was one reason for involving personnel from the investigated emergency departments as observers in this study. Furthermore, this study was limited to the emergency department context and it would be valuable to evaluate the TEAM instrument also in other settings and, as mentioned above, with teams who are likely to have more varying levels of experience and competence.

## Conclusion

Analyzing the complexities of teamwork may be overwhelming and time-intensive and validated instruments can provide the structure that observers need for discriminating between performance levels and measuring progress. The Swedish version of the TEAM instrument was evaluated at two emergency departments which showed that it offers a reliable and valid tool for measuring teamwork. The evaluation was based on real-time observations of authentic emergency teams indicating that TEAM can contribute with a useful tool for investigating teamwork in clinical settings.

## Data Availability

The datasets analyzed during the current study are available from the corresponding author on reasonable request.

## Footnotes

1 http://medicalemergencyteam.com/
